# Pattern and determinants of HIV research productivity in sub-Saharan Africa: bibliometric analysis of 1981 to 2009 PubMed papers

**DOI:** 10.1186/1471-2334-10-47

**Published:** 2010-03-05

**Authors:** Olalekan A Uthman

**Affiliations:** 1 Department of Public Health, Epidemiology, and Biostatistics, University of Birmingham, Birmingham, B15 2TT, UK

## Abstract

**Background:**

Several bibliometric studies have been published on AIDS. The findings obtained from these studies have provided a general picture of the history and growth of AIDS literature. However, factors related to variation in HIV research productivity in sub-Saharan Africa have not been examined. Therefore, this study aims to fill some of the gap in existing research to provide insights into factors associated with HIV research productivity in sub-Saharan Africa.

**Methods:**

A bibliometric analysis regarding sub-Saharan Africa HIV/AIDS research was conducted in the PubMed database for the period of 1981 to 2009. The numbers of HIV research articles indexed in PubMed was used as surrogate for total HIV research productivity. Series of univariable and multivariable negative binomial regression models were used to explore factors associated with variation in HIV research productivity in sub-Saharan Africa.

**Results:**

First authors from South Africa, Uganda and Kenya contributed almost half of the total number of HIV articles indexed in PubMed between 1981 and 2009. Uganda, Zimbabwe and Malawi had better records when the total production was adjusted for gross domestic product (GDP). Comoros, the Gambia and Guinea-Bissau were the most productive countries when the total products were normalized by number of people with HIV. There were strong positive and statistically significant correlation between countries number of indexed journal (Pearson correlation r = 0.77, p = .001), number of higher institutions (r = 0.60, p = .001), number of physicians (r = 0.83, p = .001) and absolute numbers of HIV articles.

**Conclusions:**

HIV research productivity in Africa is highly skewed. To increase HIV research output, total expenditure on health (% of GDP), private expenditure on health, and adult literacy rate may be important factors to address.

## Background

Sub-Saharan Africa is more heavily affected by Human immunodeficiency virus (HIV) and acquired immunodeficiency syndrome (AIDS) than any other region of the world[1]. Estimated 22.5 million people were living with HIV at the end of 2007 and approximately 1.7 million additional people were infected with HIV during that year[1]. In just the past year, the AIDS epidemic in Africa has claimed the lives of an estimated 1.6 million people in this region. More than 11 million children have been orphaned by AIDS. The rate of growth of AIDS literature has been reported to be of exponential nature [2,3]. This growth is similar to rapid increase in number of reported cases of AIDS since it was first published in 1981[3]. Several bibliometric studies have been published on AIDS [4-12]. These studies have evolved from descriptive, quantitative analyses of AIDS literature [4,5], to more qualitative subject and citation analyses. The findings obtained from these studies have provided a general picture of the history and growth of AIDS literature from the unsettled nature of the terminology during the early 1980s[6], to a more structured and controlled Medical Subject Headings (MeSH) terminology [7-12]. Through citation studies, clusters of topics representing networks and maps of AIDS have been obtained[13], and leading institutions and scientists have been identified[14]. However, factors related to variation in HIV research productivity in sub-Saharan Africa have not been examined. Therefore, this study aims to fill some of the gap in existing research to provide insights into factors associated with HIV research productivity in sub-Saharan Africa.

## Methods

### Data sources

PubMed database[15] was searched to obtain research volume of each countries from sub-Saharan Africa from January 1, 1981 to October 31, 2009. The numbers of HIV research articles indexed in PubMed was used as surrogate for total HIV research productivity. Articles originating from each country and published between 1981 and 2009 were generated by selecting the advanced-search option and then selecting the "publication date" field. Next, the "affiliation" field was searched for each country. The names of the countries were imputed in their different possible forms: Côte d'Ivoire and Ivory Coast for Côte d'Ivoire, and both Swasiland and Swaziland for Swaziland, for example. Some names of countries are also names of parts of other countries: Benin and Niger, for example are name of a place in Nigeria. To avoid errors arising from this, appropriate commands were used [i.e. (Niger [AD] NOT Nigeria)]. See additional file 1 for full search strategy. Though PubMed has been widely used for bibliometric analysis, it is important note that PubMed consist largely of English-language journals therefore possibly contributing to selection bias due to language barriers. In addition, PubMed do not represent all scientific and biomedical journals published. Many articles of biomedical importance appears in journals other than those included in the searched categories. Other limitations include the incorrect citation of origin for the authors, and definition of research production. By using the author addresses listed in the bylines of research articles, one can only identify countries and organizations where the authors were employed when the research was done or where the article was written, or both. Institutionally co-authored research articles co-publications are useful and tangible proxies of research involving African scientists and scholars.

Data on number of people living with HIV, adult literacy rate, gross domestic product (GDP), public expenditure on education (% of GDP), researcher and development researchers, number of higher institutions, number of indexed journal in MEDLINE, physicians (per 100,000 population), total expenditure on health, and private expenditure on health were obtained from the reports published by the United Nations Development Programs[16], World health Organization[17], and Joint United Nations Programme on HIV/AIDS[18].

### Statistical analyses

Series of univariable and multivariable negative binomial regression models were used to explore factors associated with variation in HIV research productivity in sub-Saharan Africa. The study deployed negative binomial regression, rather than Poisson regression, because there was significant evidence of over dispersion. Negative binomial regression, employing a robust method and has been shown to be to address the failure of Poisson regression model in presence of overdispersion by adding a parameter that reflects unobserved heterogeneity among observations[19].

The univariate negative binomial probability distribution of Y is:

where Γ is the gamma function.

The factor change in the rate of HIV productive was calculated from:

where *E*(*y *| *x, x*_*κ*_) is the expected count for a given **x**, and *E*(*y *| *x*_*κ *_+ *δ*) is the expected count after increasing *x*_*κ *_by *δ*. The percentage changes in the expected HIV literature production count were calculated from:

To allow for weighted comparison among the countries of origin we calculated the ratio of the number of publications from a certain country to the GDP, total expenditure on health, expenditure on education, adult literacy rate, and number of people with HIV. Finally, the Pearson correlation analysis method was used to examine the association between HIV research productive and the selected indicators. For correlation analysis, indicators were log transformed to linearise these associations. Data were processed and analyzed with Stata 10 software (Stata Corp., College Station, TX, USA).

## Results

A total of 24,487 articles indexed by PubMed within period of January 1981 to October 2009 were described in this study. Table 1 shows summary statistics for all the factors considered in this study. The number of people living with HIV by the end of 2007 ranged from as low as 200 in Comoros to as much as 5.7 million people in South Africa. As for gross domestic product (GDP) per capita, Comoros emerged as the most affluent country with value of US$ 714, whilst by contrast Burundi was the most deprived (US$ 115). The adult literacy rate ranged from 24% in Burundi to as much as 92% in Seychelles. The median (range) of private expenditure on health was 47.9 (13.4 to 87.7).

**Table 1:** Descriptive statistics of selected country-level variables

Indicator	Year	Median	Range
HIV patients (× 1000)	2007	140.0	0.2, 5700.0
Gross domestic product (US dollar)	2007	646.0	115, 19552.0
Education			
Adult literacy rate	2001-2005	67.2	23.6, 91.8
Public expenditure on education*	2000 - 2007	17.4	4.0, 29.8
Research and development researchers (× 1000)		5.0	0.0, 100
Number of high institutions**	2009	3.0	0.0, 91.0
Number of indexed journals	2009	1.0	0.0, 99.0
Health			
Physicians per 100,000 population	2002-2005	554.0	81.0, 34923.0
Total expenditure on health***	2006	4.9	1.5, 12.3
Private expenditure on health	2006	47.9	13.4, 87.7

* Percentage of GDP

** Higher institutions - universities, colleges and polytechnics

*** Percentage of total expenditure on health

**Figure 1:**
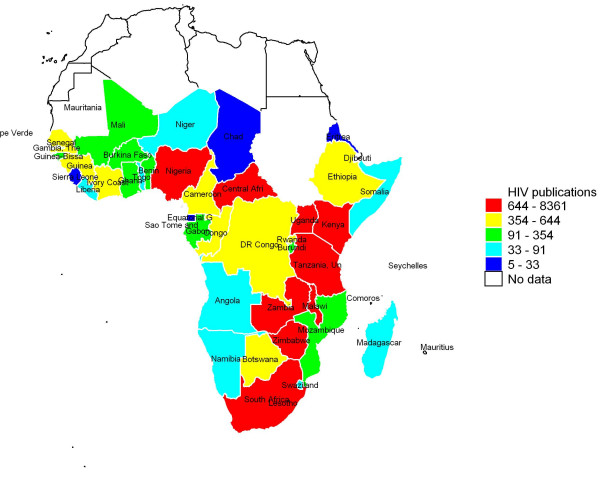
**Color-coded map representing number HIV research articles indexed in PubMed (1981 - 2009)**.

Table 2 shows the top 10 top-ranking countries in terms of relative contribution of each country to the total number of articles that were index by PubMed (see additional file 2 full list). First authors from South Africa, Uganda and Kenya produced highest number of articles. Figure 1 shows number of HIV publications broken down by quintiles. Nine countries had more than 640 publications that put them in the highest quintile, nine belong in the second quintile (354 to 644 publications), nine in third quintile (91 to 354 publications), nine in the fourth quintile (33 to 91 publications), and ten countries in the lowest quintile with five to 33 publications. Table 2 also make it abundantly clear that first authors from South Africa, Uganda and Kenya had largest number of HIV publications indexed in PubMed even after adjustment were made for total expenditure on health, expenditure on education, and adult literacy rate. However, when the total product was adjusted for GDP, countries Uganda, Zimbabwe and Malawi were the most productive countries (Table 3). Whereas countries such as South Africa and Nigeria had a lower number of PubMed publications relative to their GDP.

**Table 2:** Number of medical publications in HIV/AIDS in relation to indicators, sub-Saharan Africa, 1981-2009

	Total publications		PUBLICATIONS per				
		
Rank	Country	Number (%)	Gross Domestic Product	Total expenditure on health	Expenditure on education	Adult literacy rate	HIV patients
**1**	South Africa	8361 (34.1)	Uganda	South Africa	South Africa	South Africa	Comoros
**2**	Uganda	1987 (8.1)	Zimbabwe	Kenya	Uganda	Uganda	Gambia
**3**	Kenya	1778 (7.3)	Malawi	Congo	Kenya	Kenya	Guinea-Bissau
**4**	Tanzania	1198 (4.9)	DR Congo	Uganda	Congo	Guinea	Guinea
**5**	Nigeria	1120 (4.6)	Tanzania	Nigeria	Zambia	Tanzania	Senegal
**6**	Zimbabwe	1045 (4.3)	Kenya	Tanzania	Cameroon	Nigeria	Congo
**7**	Zambia	922 (3.8)	Ethiopia	Zambia	Ivory coast	Ethiopia	Madagascar
**8**	Malawi	890 (3.6)	South Africa	Ivory coast	Guinea	Malawi	Gabon
**9**	Guinea	640 (2.6)	Guinea	Zimbabwe	Ethiopia	Zambia	Rwanda
**10**	Congo	639 (2.6)	Rwanda	Guinea	Rwanda	Ivory coast	Equatorial Guinea

**Table 3:** Factors associated with HIV research productivity in sub-Saharan Africa, identified by negative binomial regressions

	Univariable		Multivariable	
	
Indicator^†^	Coefficient (SE)	Percent change	Coefficient (SE)	Percent change
HIV patients	0.59(0.06)***	80.2	0.51(0.10)***	66.1
Gross domestic product	ns	ns	ns	ns
Education				
Adult literacy rate	1.33(0.50)**	279.8	ns	ns
Public expenditure on education	ns	ns	ns	ns
Research and development researchers	0.54(0.22)*	71.7	ns	ns
Number of high institutions	0.70(0.14)***	101.7	ns	ns
Number of indexed journals	0.88(0.11)***	141.2	0.59(0.16)***	117.2
Health				
Physicians per 100,000 population	0.73(0.11)***	108.2	ns	ns
Total expenditure on health	1.55(0.41)***	373.2	1.01(0.29)*	173.9
Private expenditure on health	1.70(0.47)***	445.0	0.60(0.29)*	81.4

SE-standard error; ns-not significant; * p <.05, **p < .01, ***p < 0.001

^†^Indicators were log-transformed

**Figure 2:**
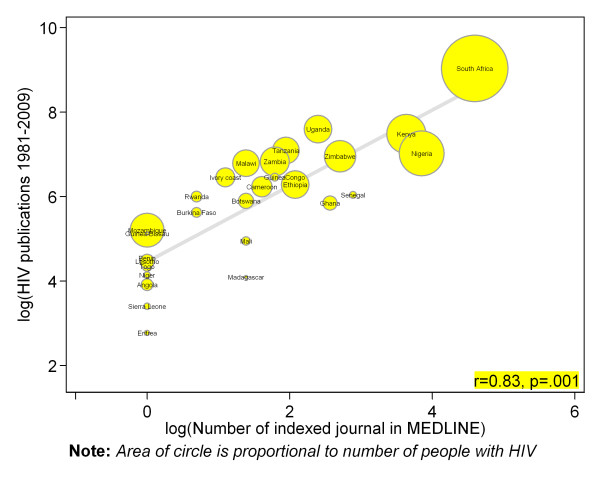
**Scatter plot depicting the association between total PubMed publications for different countries in sub-Saharan Africa and number of index journal in MEDLINE**.

**Figure 3:**
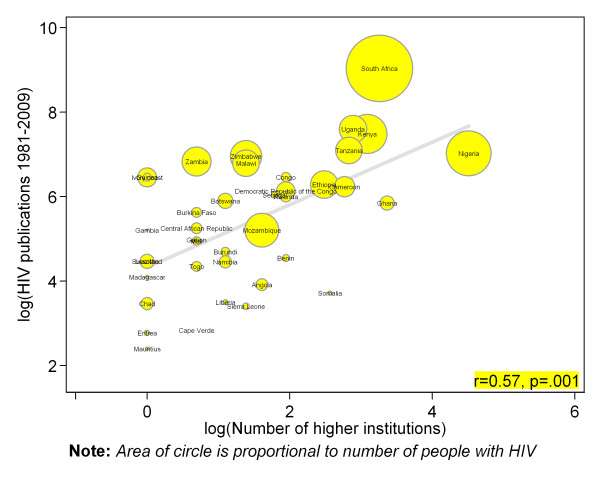
**Scatter plot depicting the association between total PubMed publications for different countries in sub-Saharan Africa and number of higher institutions**.

**Figure 4:**
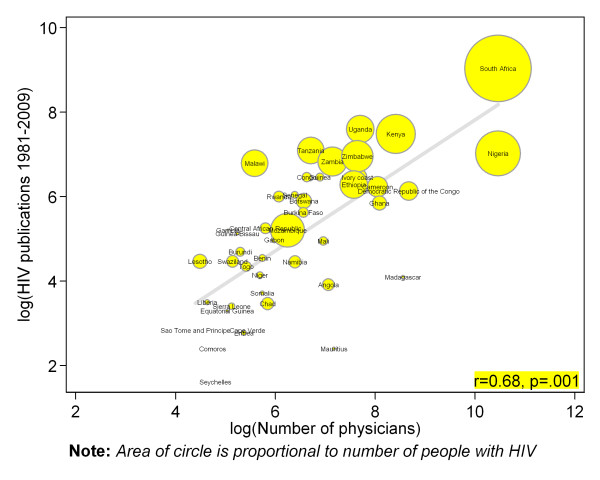
**Scatter plot depicting the association between total PubMed publications for different countries in sub-Saharan Africa and number of physicians**.

**Figure 5:**
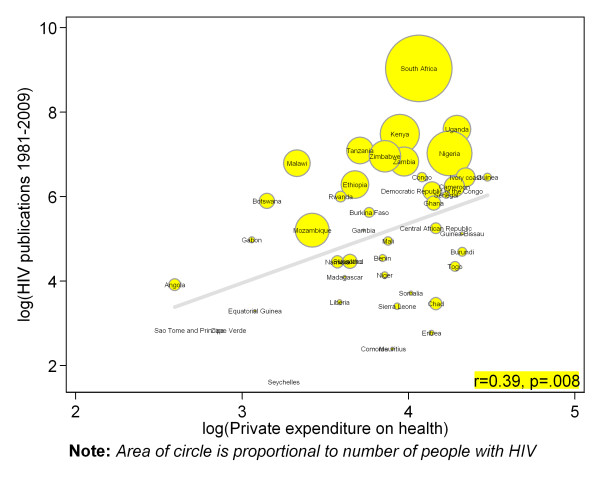
**Scatter plot depicting the association between total PubMed publications for different countries in sub-Saharan Africa and private expenditure on health**.

There were strong positive and statistically significant correlation between countries number of indexed journal (Pearson correlation r = 0.83, p = .001) (figure 2), number of higher institutions (r = 0.57, p = .001) (figure 3), number of physicians (r = 0.68, p = .001) (figure 4) and absolute numbers of HIV articles. As shown in figure 5, there was a moderate positive and statistically significant correlation between PubMed HIV publications and country's private expenditure on health (r = 0.40, p = .008). Results of univariable negative binomial regression analyses are shown in Table 3. Number of people living with HIV (beta-coefficient [β] 0.59=, p = .001), adult literacy rate (β= 1.33, p = .011), research and development researchers (β = 0.54, p = .013), number of higher institutions (β= 0.70, p = .001), number of indexed journals (β = 0.88, p = .001), number of physicians (β = 0.73, p = .001), total expenditure on health (β = 1.55, p = .001), and private expenditure on health (β = 1.70, p = .001) emerged as significant factors. Table 3 also shows results of multivariable analysis when all factors were controlled for statistically. Only number of people living with HIV (beta-coefficient [β] = 0.51, p = .001), number of indexed journals (0.59, p = .001), total expenditure on health (1.01, p = .017), and private expenditure on health (0.60, p = .040) remained significantly associated with HIV publications.

## Discussion

The study shows that HIV research productivity in SSA is highly skewed; South Africa, Uganda, and Kenya jointly account for almost 50% of all HIV/AIDS-related publications in HIV/AIDS indexed in PubMed between 1981 and 2009. This finding is consistent with results from other studies [11,20,21]. This study found like others, that the better economic ranking of a country the higher the quantity of itsresearch productivity [20-22]. This study also echoes findings of a previous study that found Malawi tended to most productive when the total HIV publications was normalized by GDP[11]. Falagas and colleagues [8] reviewed the medical literature in order to evaluate the contribution of different world regions on HIV/AIDS research indexed in the Journal Citation Reports (JCR) and the Web of Science databases of the Institute for Scientific Information (ISI). A total of 9502 articles on HIV/AIDS were retrieved from three AIDS journals over an 18-year study period [8]. Falagas et.al. [8] reported that the United States and Western Europe made up a striking 83% and 92% of the world's research production on HIV/AIDS, respectively [8]. About half of articles originating in Latin America and the Caribbean and half in Asia were produced in collaboration with the United States [8]. Only 40% of articles from Africa and 58% from Eastern Europe were produced in cooperation with Western Europe. Falagas et al [8] concluded that collaboration between researchers within developing regions was negligible. Another study recovered a total of 11 826 papers dealing with HIV/AIDS included in the ISI databases in 2003, and found that the leading countries according to the total number of publications were the USA, UK, and France [11]. A bibliometric study regarding AIDS in Latin America and the Caribbean for the period 1980 to 1996 found that while Haiti was the most productive country in the region [10]. Uthman [12] analysed the trends in Nigeria's SCI publications in HIV/AIDS from 1980 to 2006. Uthman [12] found that Nigeria has achieved a significant increase in the number of SCI publications and collaborations in HIV literature and over 85% of the articles were published in collaboration among two or more authors [12]. The USA, as the most important collaborating partner of Nigeria's HIV/AIDS researchers, contributed 30.8% of articles with international collaboration [12].

Evidence from univariable negative binomial regression models suggests that that adult literacy rate, total expenditure on health, and private expenditure on health are the most important predictor of HIV research productivity in sub-Saharan Africa.

For every one log increase in adult literacy rate, the predicted mean HIV productivity increased by 280%. For every one percent of GDP committed to health and one log increase in private expenditure on health, the predicted mean HIV productivity increased by 373% and 445% respectively. Based on the based possible estimates from multivariable negative binomial regression, number of people with HIV, number of indexed journals, total expenditure on health, and private expenditure on health are the main significant factors associated with HIV research productivity in sub-Saharan Africa. A similar finding has been reported previously [23,24]. Rahman and Fukui studied factors related to worldwide variation in biomedical research productivity[23]. Rahman and Fukui found that gross national product (GNP) per capita and research and development (R&D) expenditure were statistically significantly associated with biomedical research productivity[23]. Another study found that that low gross national product per capita, insufficient number of physicians, and inadequate public spending on the health sector were responsible for the meager number of biomedical publications in Asian countries [24].

## Conclusions

In summary, this study examined almost three decades of HIV literature production by first authors from sub-Saharan Africa. The results of the study showed that South Africa, Nigeria, and Uganda were the most productive countries in terms of absolute number of publications indexed by PubMed through the period studied. Based on best possible estimates, number of people with HIV, number of indexed journals, total expenditure on health, and private expenditure on health are the main factors related to HIV research productivity in sub-Saharan Africa.

## Competing interests

The author declares that they have no competing interests.

## Authors' contributions

OAU conceived the study, extracted the data, did the analyses and interpretation, and wrote the first draft of the manuscript.

## Pre-publication history

The pre-publication history for this paper can be accessed here:

http://www.biomedcentral.com/1471-2334/10/47/prepub

## Supplementary Material

Additional file 1 Full search strategy. Click here for file

Additional file 2 Number of medical publications in HIV/AIDS in relation to indicators, sub-Saharan Africa, 1981-2009. Click here for file
