# Supplementary material for: Pattern and determinants of HIV research productivity in sub-Saharan Africa: bibliometric analysis of 1981 to 2009 PubMed papers
Source: BMC Infect Dis. 2010 Mar 5;10:47. doi: 10.1186/1471-2334-10-47 (PMC2841182; doi:10.1186/1471-2334-10-47)
Supplement: Additional file 1 — Full search strategy. [file 1471-2334-10-47-S1.PDF]

PubMed Search Stragies (October 2009)

Search	Most Recent Queries	Result
#47	Search **Zimbabwe OR Zimbabwe[PL] OR Zimbabwe[AD] OR Zimbabwe[TI] AND #1 AND #2**	1045
#46	Search **Zambia OR Zambia[PL] OR Zambia[AD] OR Zambia[TI] AND #1 AND #2**	922
#45	Search **United Republic of Tanzania OR United Republic of Tanzania[PL] OR United Republic of Tanzania[AD] OR United Republic of Tanzania[TI] OR TANZANIA OR TANZANIA [PL] OR TANZANIA [AD] OR TANZANIA [TI] AND #1 AND #2**	1198
#44	Search **Uganda OR Uganda[PL] OR Uganda[AD] OR Uganda[TI] AND #1 AND #2**	1987
#43	Search **Togo OR Togo[PL] OR Togo[AD] OR Togo[TI] AND #1 AND #2**	77
#42	Search **Swaziland OR Swaziland [PL] OR Swaziland [AD] OR Swaziland [TI] OR Swasiland OR Swasiland [PL] OR Swasiland [AD] OR Swasiland [TI] AND #1 AND #2**	87
#41	Search **South Africa OR South Africa[PL] OR South Africa[AD] OR South Africa[TI] AND #1 AND #2**	8361
#40	Search **Somalia OR Somalia[PL] OR Somalia[AD] OR Somalia[TI] AND #1 AND #2**	41
#39	Search **Sierra Leone OR Sierra Leone[PL] OR Sierra Leone[AD] OR Sierra Leone[TI] AND #1 AND #2**	30
#38	Search **Seychelles OR Seychelles[PL] OR Seychelles[AD] OR Seychelles[TI] AND #1 AND #2**	5
#37	Search **Senegal OR Senegal[PL] OR Senegal[AD] OR Senegal[TI] AND #1 AND #2**	419
#36	Search **Sao Tome and Principe OR Sao Tome and Principe[PL] OR Sao Tome and Principe[AD] OR Sao Tome and Principe[TI] AND #1 AND #2**	17
#35	Search **Rwanda OR Rwanda[PL] OR Rwanda[AD] OR Rwanda[TI] AND #1 AND #2**	402
#34	Search **Nigeria OR Nigeria[PL] OR Nigeria[AD] OR Nigeria[TI] AND #1 AND #2**	1120
#33	Search **((Niger OR Niger[PL] OR Niger[AD] OR Niger[TI]) NOT (Nigeria OR Nigeria[PL] OR Nigeria[AD] OR Nigeria[TI])) AND #1 AND #2**	63
#32	Search **Namibia OR Namibia[PL] OR Namibia[AD] OR Namibia[TI] AND #1 AND #2**	86
#31	Search **Mozambique OR Mozambique[PL] OR Mozambique[AD] OR Mozambique[TI] AND #1 AND #2**	182
#30	Search **Mauritius OR Mauritius[PL] OR Mauritius[AD] OR Mauritius[TI] AND #1 AND #2**	11
#29	Search **Mali OR Mali[PL] OR Mali[AD] OR Mali[TI] AND #1 AND #2**	141

#28	Search **Malawi OR Malawi[PL] OR Malawi[AD] OR Malawi[TI] AND #1 AND #2**	890
#27	Search **Madagascar OR Madagascar[PL] OR Madagascar[AD] OR Madagascar[TI] AND #1 AND #2**	60
#26	Search **Liberia OR Liberia[PL] OR Liberia[AD] OR Liberia[TI] AND #1 AND #2**	33
#25	Search **Lesotho OR Lesotho[PL] OR Lesotho[AD] OR Lesotho[TI] AND #1 AND #2**	87
#24	Search **Kenya OR Kenya[PL] OR Kenya[AD] OR Kenya[TI] AND #1 AND #2**	1778
#23	Search **Guinea-Bissau OR Guinea-Bissau[PL] OR Guinea-Bissau[AD] OR Guinea-Bissau[TI] AND #1 AND #2**	168
#22	Search **Guinea OR Guinea[PL] OR Guinea[AD] OR Guinea[TI] AND #1 AND #2**	640
#21	Search **Ghana OR Ghana[PL] OR Ghana[AD] OR Ghana[TI] AND #1 AND #2**	345
#20	Search **Gambia OR Gambia[PL] OR Gambia[AD] OR Gambia[TI] AND #1 AND #2**	182
#19	Search **Gabon OR Gabon[PL] OR Gabon[AD] OR Gabon[TI] AND #1 AND #2**	145
#18	Search **Ethiopia OR Ethiopia [PL] OR Ethiopia [AD] OR Ethiopia [TI] AND #1 AND #2**	536
#17	Search **Eritrea OR Eritrea [PL] OR Eritrea [AD] OR Eritrea [TI] AND #1 AND #2**	16
#16	Search **Equatorial Guinea OR Equatorial Guinea [PL] OR Equatorial Guinea [AD] OR Equatorial Guinea [TI] AND #1 AND #2**	27
#15	Search **ZAIRE OR ZAIRE [AD] OR ZAIRE (PL) OR Democratic Republic of the Congo OR Democratic Republic of the Congo [PL] OR Democratic Republic of the Congo [AD] OR Democratic Republic of the Congo[TI] OR DR CONGO [AD] AND #1 AND #2**	459
#14	Search **Côte d'Ivoire OR Côte d'Ivoire [PL] OR Côte d'Ivoire [AD] OR Côte d'Ivoire [TI] OR ivory coast OR ivory coast [PL] OR ivory coast [AD] OR ivory coast[TI] AND #1 AND #2**	636
#13	Search **Congo OR CONGO[PL] OR CONGO[AD] OR Comoros [TI] AND #1 AND #2**	639
#12	Search **Comoros OR Comoros [PL] OR Comoros [AD] OR Comoros [TI] AND #1 AND #2**	11
#11	Search **Chad OR Chad [PL] OR Chad [AD] OR CHAD [TI] AND #1 AND #2**	32
#10	Search **Central African Republic OR Central African Republic [PL] OR Central African Republic [AD] OR Central African Republic [TI] AND #1 AND #2**	190
#9	Search **Cape Verde OR Cape Verde [PL] OR Cape Verde [AD] OR Cape Verde [TI] AND #1 AND #2**	17
#8	Search **Cameroon OR Cameroon [PL] OR Cameroon [AD] OR Cameroon [TI] AND #1 AND #2**	509

#7	Search **Burundi OR Burundi [PL] OR Burundi [AD] OR Burundi [TI] AND #1 AND #2**	109
#6	Search **Burkina Faso OR Burkina Faso[PL] OR Burkina Faso[AD] OR Burkina Faso[TI] AND #1 AND #2**	277
#5	Search **Botswana OR Botswana [PL] OR Botswana [AD] OR Botswana[TI] AND #1 AND #2**	363
#4	Search **(Benin OR Benin[PL] OR Benin[AD] OR BENIN [TI]) NOT (Nigeria OR Nigeria[PL] OR Nigeria[AD] OR Nigeria[TI]) AND #1 AND #2**	94
#3	Search **Angola OR Angola [PL] OR Angola [AD] OR ANGOLA [TI] AND #1 AND #2**	50
#2	Search **HIV Infections[MeSH] OR HIV[MeSH] OR hiv[tw] OR hiv-1*[tw] OR hiv-2*[tw] OR hiv1[tw] OR hiv2[tw] OR hiv infect*[tw] OR human immunodeficiency virus[tw] OR human immunedeficiency virus[tw] OR human immuno-deficiency virus[tw] OR human immune-deficiency virus[tw] OR ((human immun*) AND (deficiency virus[tw])) OR acquired immunodeficiency syndrome[tw] OR acquired immunedeficiency syndrome[tw] OR acquired immuno-deficiency syndrome[tw] OR acquired immune-deficiency syndrome[tw] OR ((acquired immun*) AND (deficiency syndrome[tw]))** Limits: **Publication Date from 1981**	249683
#1	Search Limits: **Publication Date from 1981**	13591259
