# Supplementary material for: Pattern and determinants of HIV research productivity in sub-Saharan Africa: bibliometric analysis of 1981 to 2009 PubMed papers
Source: BMC Infect Dis. 2010 Mar 5;10:47. doi: 10.1186/1471-2334-10-47 (PMC2841182; doi:10.1186/1471-2334-10-47)
Supplement: Additional file 2 — Number of medical publications in HIV/AIDS in relation to indicators, sub-Saharan Africa, 1981-2009. [file 1471-2334-10-47-S2.PDF]

List of countries and their HIV publications indexed in PubMed, 1981-2009

Rank	Country	Total publication
1	South Africa	8361
2	Uganda	1987
3	Kenya	1778
4	Tanzania	1198
5	Nigeria	1120
6	Zimbabwe	1045
7	Zambia	922
8	Malawi	890
9	Central African Republic	647
10	Guinea	640
11	Congo	639
12	Ivory coast	636
13	Ethiopia	536
14	Cameroon	509
15	Democratic Republic of the Congo	459
16	Senegal	419
17	Rwanda	402
18	Botswana	363
19	Ghana	345
20	Burkina Faso	277
21	Gambia	182
22	Mozambique	182
23	Guinea-Bissau	168
24	Gabon	145
25	Mali	141

Rank	Country	Total publication
26	Burundi	109
27	Benin	94
28	Swaziland	87
29	Lesotho	87
30	Namibia	86
31	Togo	77
32	Niger	63
33	Madagascar	60
34	Angola	50
35	Somalia	41
36	Liberia	33
37	Chad	32
38	Sierra Leone	30
39	Equatorial Guinea	27
40	Cape Verde	17
41	Sao Tome and Principe	17
42	Eritrea	16
43	Mauritius	11
44	Comoros	11
45	Seychelles	5

Number of medical publications in HIV/AIDS in relation to gross domestic product (GDP), sub-Saharan Africa, 1981-2009

Rank	Country	Publication / GDP
1	Uganda	5.2152
2	Zimbabwe	4.0038
3	Malawi	3.4766
4	Democratic Republic of the Congo	3.2098
5	Tanzania	2.9950
6	Kenya	2.7566
7	Ethiopia	2.1878
8	Central African Republic	1.6421
9	South Africa	1.4138
10	Guinea	1.3142
11	Rwanda	1.1720
12	Nigeria	1.0018
13	Zambia	0.9675
14	Burundi	0.9478
15	Guinea-Bissau	0.7962
16	Ivory coast	0.6193
17	Burkina Faso	0.6048
18	Ghana	0.5341
19	Mozambique	0.5000
20	Gambia	0.4828
21	Senegal	0.4656
22	Cameroon	0.4561
23	Congo	0.3148

Rank	Country	Publication / GDP
24	Mali	0.2536
25	Niger	0.2143
26	Togo	0.2026
27	Liberia	0.1667
28	Madagascar	0.1600
29	Benin	0.1564
30	Lesotho	0.1090
31	Sierra Leone	0.1056
32	Eritrea	0.0563
33	Botswana	0.0555
34	Chad	0.0486
35	Swaziland	0.0345
36	Namibia	0.0255
37	Sao Tome and Principe	0.0186
38	Gabon	0.0167
39	Comoros	0.0154
40	Angola	0.0138
41	Cape Verde	0.0063
42	Mauritius	0.0020
43	Equatorial Guinea	0.0014
44	Seychelles	0.0006

Number of medical publications in HIV/AIDS in relation total expenditure on health, sub-Saharan Africa, 1981-2009

Rank	Country	Publications per total expenditure on health
1	South Africa	972.21
2	Kenya	386.52
3	Congo	304.29
4	Uganda	275.97
5	Nigeria	273.17
6	Tanzania	217.82
7	Zambia	177.31
8	Ivory coast	167.37
9	Central African Republic	165.90
10	Zimbabwe	124.40
11	Guinea	112.28
12	Ethiopia	109.39
13	Democratic Republic of the Congo	106.74
14	Cameroon	97.88
15	Senegal	77.59
16	Malawi	72.36
17	Ghana	55.65
18	Botswana	50.42
19	Burkina Faso	43.28
20	Gambia	42.33
21	Gabon	39.19
22	Mozambique	38.72
23	Rwanda	38.65

Rank	Country	Publications per total expenditure on health
24	Burundi	36.33
25	Guinea-Bissau	27.10
26	Mali	23.50
27	Madagascar	18.75
28	Angola	18.52
29	Equatorial Guinea	18.00
30	Benin	17.74
31	Namibia	17.55
32	Somalia	15.77
33	Niger	15.75
34	Swaziland	14.75
35	Togo	14.00
36	Lesotho	12.99
37	Chad	8.89
38	Sierra Leone	8.57
39	Liberia	5.89
40	Eritrea	3.56
41	Comoros	3.44
42	Cape Verde	3.04
43	Mauritius	2.56
44	Sao Tome and Principe	1.62
45	Seychelles	0.74

Number of medical publications in HIV/AIDS in relation expenditure on education, sub-Saharan Africa, 1981-2009

Rank	country	Publications per expenditure on education
1	South Africa	480.52
2	Uganda	108.58
3	Kenya	99.33
4	Congo	78.89
5	Zambia	62.30
6	Cameroon	29.94
7	Ivory coast	29.58
8	Guinea	25.00
9	Ethiopia	23.00
10	Rwanda	21.16
11	Gambia	20.45
12	Burkina Faso	17.99
13	Botswana	17.29
14	Senegal	15.93
15	Mozambique	8.67
16	Mali	8.39
17	Equatorial Guinea	6.75
18	Burundi	6.16
19	Togo	5.66
20	Benin	5.50
21	Namibia	4.10
22	Madagascar	3.66
23	Niger	3.58

Rank	country	Publications per expenditure on education
24	Chad	3.17
25	Lesotho	2.92
26	Cape Verde	1.04
27	Mauritius	0.87
28	Comoros	0.46
29	Seychelles	0.40

Number of medical publications in HIV/AIDS in relation to adult literacy rate, sub-Saharan Africa, 1981-2009

Rank	Country	Publications per adult literacy rate
1	South Africa	101.47
2	Uganda	29.18
3	Kenya	24.16
4	Guinea	21.69
5	Tanzania	17.26
6	Nigeria	16.23
7	Ethiopia	14.93
8	Malawi	13.88
9	Zambia	13.56
10	Central African Republic	13.31
11	Ivory coast	13.06
12	Burkina Faso	11.74
13	Zimbabwe	11.68
14	Senegal	10.66
15	Congo	7.57
16	Cameroon	7.50
17	Democratic Republic of the Congo	6.83
18	Rwanda	6.19
19	Ghana	5.96
20	Mali	5.88
21	Mozambique	4.70
22	Botswana	4.47
23	Benin	2.71

Rank	Country	Publications per adult literacy rate
24	Niger	2.20
25	Burundi	1.84
26	Gabon	1.73
27	Togo	1.45
28	Chad	1.25
29	Swaziland	1.09
30	Lesotho	1.06
31	Namibia	1.01
32	Sierra Leone	0.86
33	Madagascar	0.85
34	Angola	0.74
35	Liberia	0.55
36	Equatorial Guinea	0.31
37	Cape Verde	0.21
38	Sao Tome and Principe	0.20
39	Mauritius	0.13
40	Seychelles	0.05

Number of medical publications in HIV/AIDS in relation to number of people with HIV, sub-Saharan Africa, 1981-2009

Rank	country	Publications per 1000 HIV patients
1	Comoros	55.00
2	Gambia	22.20
3	Guinea-Bissau	10.50
4	Guinea	7.36
5	Senegal	6.25
6	Congo	5.33
7	Madagascar	4.29
8	Central African Republic	4.04
9	Gabon	2.96
10	Rwanda	2.68
11	Equatorial Guinea	2.45
12	Burkina Faso	2.13
13	Uganda	1.99
14	Somalia	1.71
15	Benin	1.47
16	South Africa	1.47
17	Mali	1.41
18	Ghana	1.33
19	Ivory coast	1.33
20	Tanzania	1.27
21	Botswana	1.21
22	Niger	1.05
23	Democratic Republic of the Congo	1.02

Rank	country	Publications per 1000 HIV patients
24	Burundi	0.99
25	Malawi	0.96
26	Liberia	0.94
27	Cameroon	0.94
28	Kenya	0.89
29	Mauritius	0.85
30	Zambia	0.84
31	Zimbabwe	0.80
32	Togo	0.59
33	Ethiopia	0.55
34	Sierra Leone	0.55
35	Swaziland	0.46
36	Nigeria	0.43
37	Namibia	0.43
38	Eritrea	0.42
39	Lesotho	0.32
40	Angola	0.26
41	Chad	0.16
42	Mozambique	0.12
